# Supplementary material for: Oxygen Gas Sensing Using a Hydrogel-Based Organic Electrochemical Transistor for Work Safety Applications
Source: Polymers (Basel). 2022 Mar 3;14(5):1022. doi: 10.3390/polym14051022 (PMC8912781; doi:10.3390/polym14051022)
Supplement: Supplementary file 1 [file polymers-14-01022-s001.zip › polymers-1577615-supplementary.pdf]

polymers

MDPI

Supplementary Material: Gas-Oxygen Sensing Using a Hydrogel-Based Organic Electrochemical Transistor for Work Safety Applications

Francesco Decataldo, Filippo Bonafè, Federica Mariani, Martina Serafini, Marta Tessarolo, Isacco Gualandi, Erika Scavetta, Beatrice Fraboni

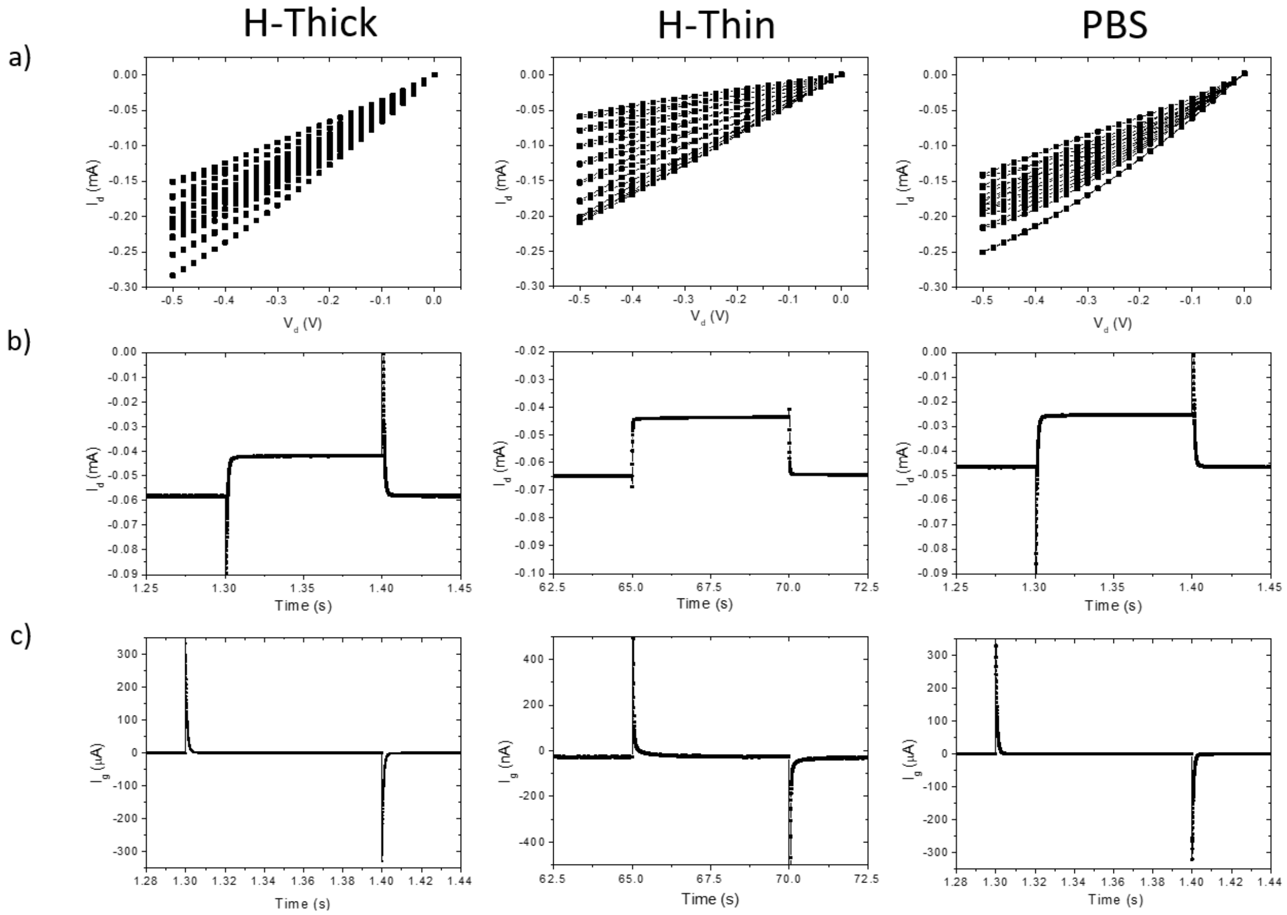

Figure S1. a) Output curves for OECT having the thick hydrogel (left), the thin one (center) or PBS (right) as electrolyte for gating. $I_d(t)$ (**b**) and $I_g(t)$ (**c**) OECT responses upon the application of a square positive voltage on the gate (+0.3V).

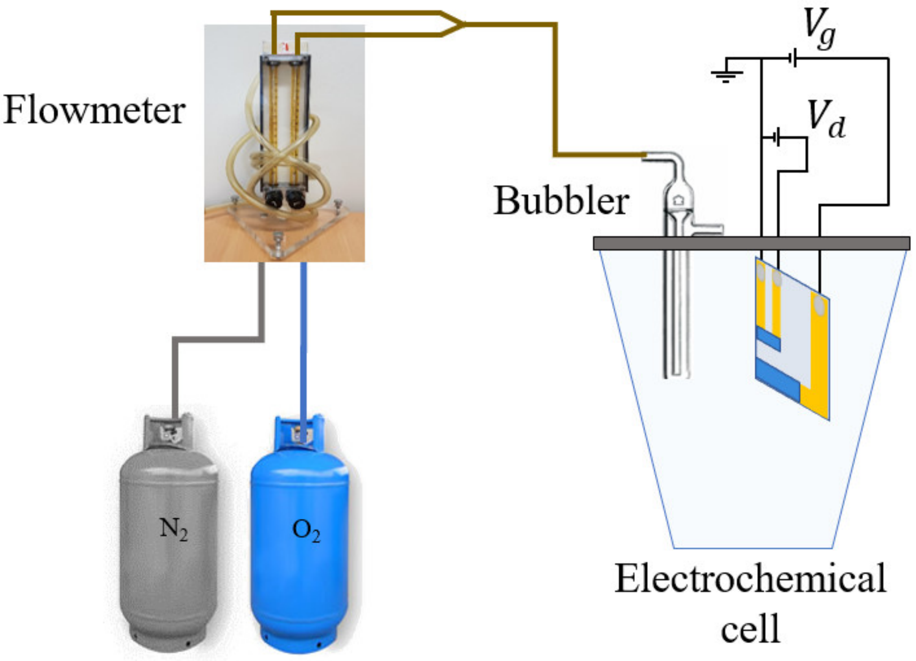

Figure S2. Experimental setup for the oxygen sensing measurements. A flowmeter is used to mix nitrogen and oxygen in the required percentage, which is then fluxed inside the sealed electrochemical cell through a bubbler.

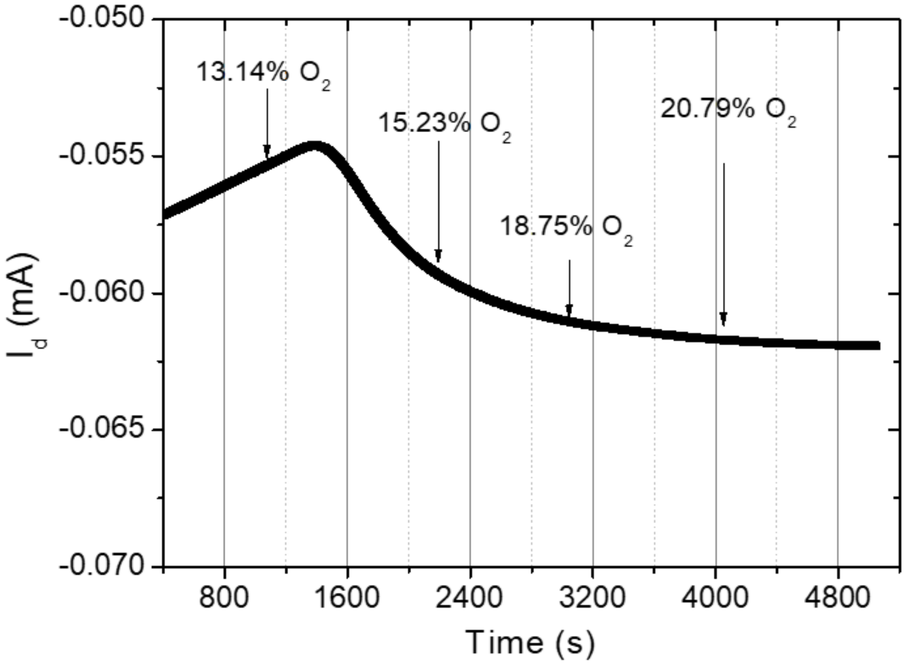

Figure S3. Oxygen sensing using a H-Thick OECT, thus having the 5mm-thick hydrogel as electrolyte.

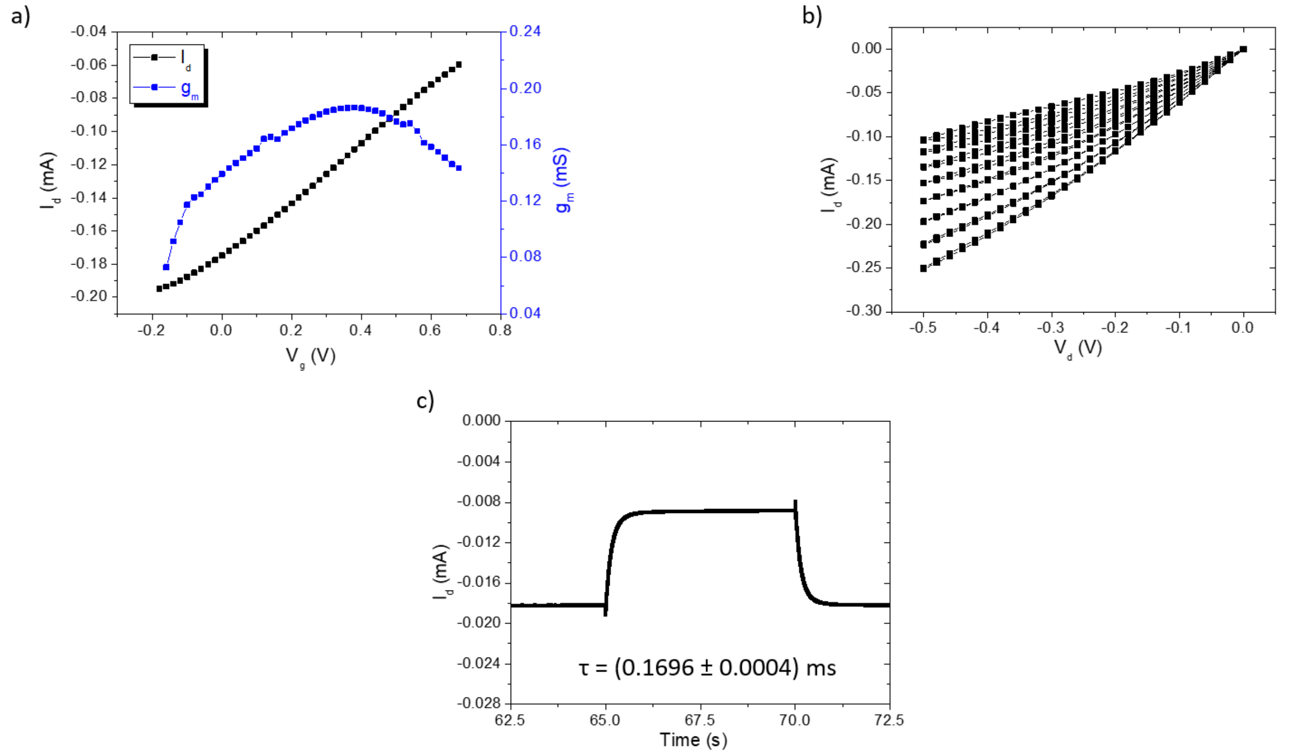

Figure S4. Glycerol-treated, H-Thin OECT electrical performances. Transcharacteristic curve (black dotted line) and relative transconductance (blue dotted line) (**a**), output (**b**) and current modulation response upon the application of a square voltage pulse (0.3V) on the gate (**c**), showing comparable values to the electrical characterization obtained without glycerol coating.

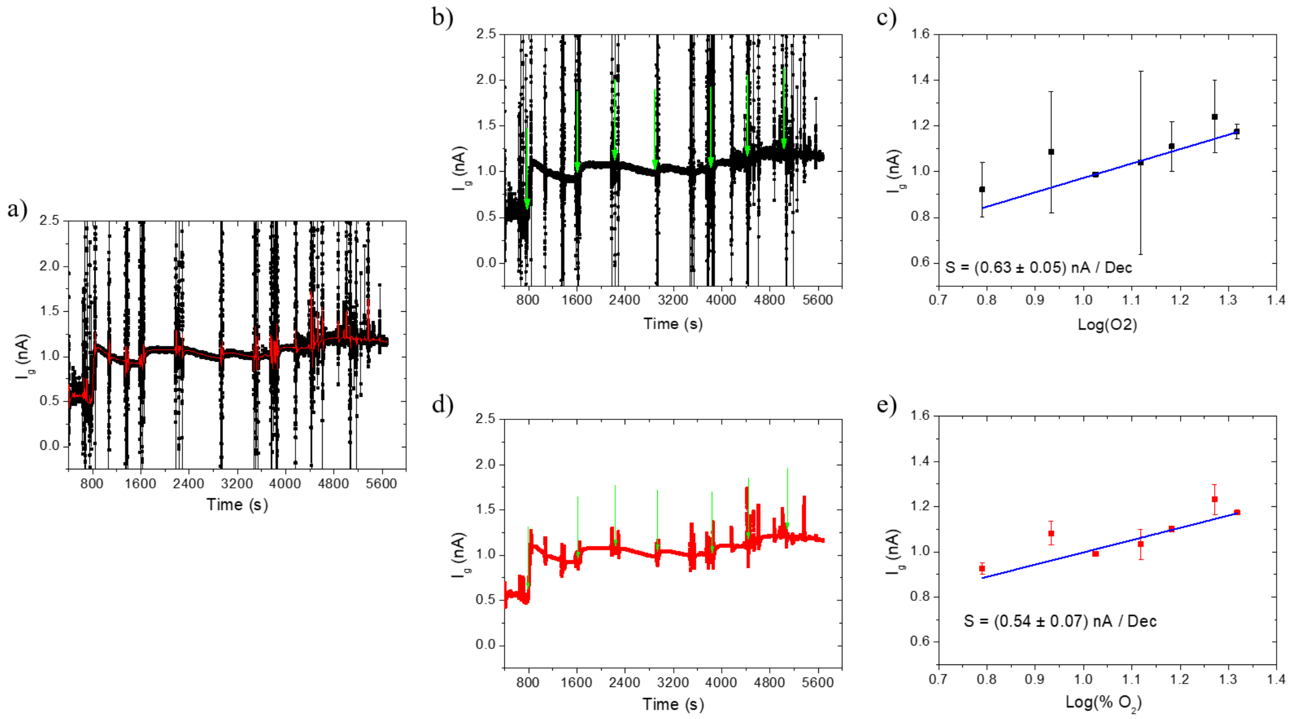

Figure S5. H-thin OECT on plastic substrate for oxygen sensing. (**a**) Bare I_g(t) measurement (black dotted line) and smoothed data (red line) upon oxygen additions in the sealed cell, starting from a nitrogen-saturated environment (0% of O_2). I_g(t) measurement (**b**) with oxygen additions (green arrows) and relative sensor calibration curve (**c**) for the bare data. I_g(t) measurement (**d**) with oxygen additions (green arrows) and relative sensor calibration curve (**e**) for the smoothed data.

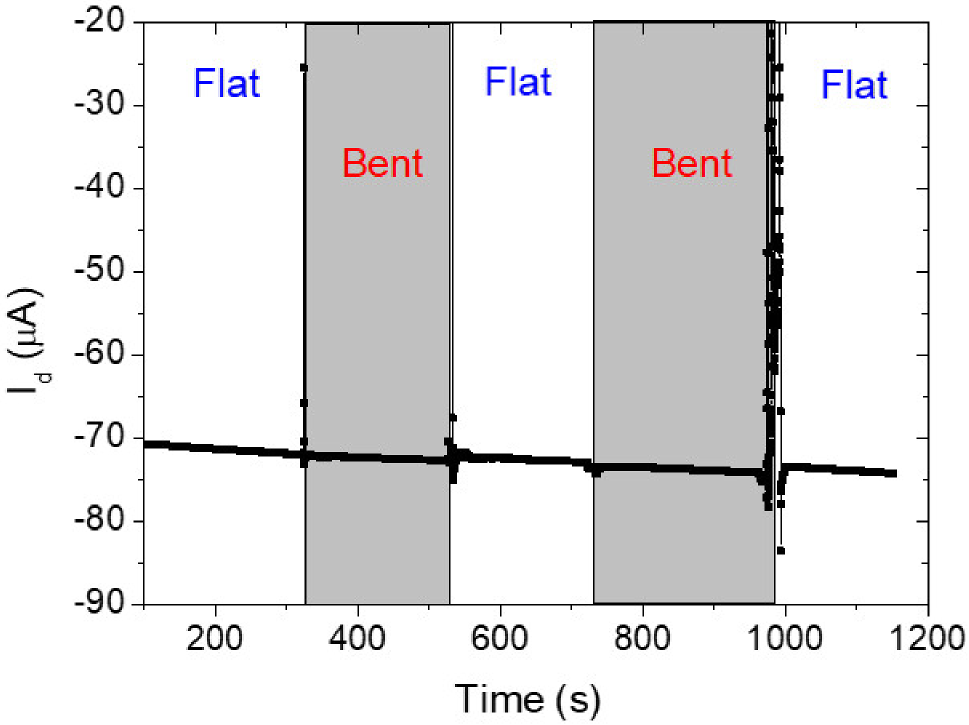

Figure S6. Repeated flat versus bent sequence test in air at constant transistor polarization (V_d =-0.3V, V_g =0.3V).
